# Recurrent DMD Deletions Highlight Specific Role of Dp71 Isoform in Soft-Tissue Sarcomas

**DOI:** 10.3390/cancers11070922

**Published:** 2019-07-01

**Authors:** Olivier Mauduit, Vanessa Delcroix, Tom Lesluyes, Gaëlle Pérot, Pauline Lagarde, Lydia Lartigue, Jean-Yves Blay, Frédéric Chibon

**Affiliations:** 1 Institut national de la santé et de la recherche médicale (INSERM) U1218, Bergonié Cancer Institute, F-33076 Bordeaux, France; 2 ED 340 BMIC, Claude Bernard Lyon 1 University, F-69622 Villeurbanne, France; 3 Department of Pathology, Bergonié Cancer Institute, F-33076 Bordeaux, France; 4 Department of Life and Health Sciences, University of Bordeaux, F-33000 Bordeaux, France; 5 Department of Pathology, Léon Bérard Center, F-69003 Lyon, France

**Keywords:** sarcoma with complex genomics, myogenic sarcoma, DMD, Dp71, cancer

## Abstract

Soft-tissue sarcomas (STS) are rare tumors whose oncogenesis remains unknown and for which no common therapeutic target has yet been identified. Analysis of 318 STS by CGH array evidenced a frequent deletion affecting the *DMD* gene (encoding dystrophin isoforms) in 16.5% of STS, including sarcomas with complex genomics, gastrointestinal tumors (GIST), and synovial sarcomas (SS). These deletions are significantly associated with metastatic progression, thus suggesting the role of DMD downregulation in the acquisition of aggressive phenotypes. We observed that targeted deletions of DMD were restricted to the 5’ region of the gene, which is responsible for the transcription of Dp427. Analysis of STS tumors and cell lines by RNA sequencing revealed that only the Dp71 isoform was widely expressed. Dp427 depletion had no effect on cell growth or migration. However, Dp71 inhibition by shRNA dramatically reduced the cell proliferation and clonogenicity of three STS cell lines, likely by altering the cell cycle progression through the G2/M-phase. Our work demonstrates that DMD deletions are not restricted to myogenic tumors and could be used as a biomarker for metastatic evolution in STS. Dp71 seems to play an essential role in tumor growth, thus providing a potential target for future STS treatments.

## 1. Introduction

DMD is one of the longest genes in the human genome, spanning 2.2 Mb [1]. It is composed of 79 exons coding for a full-length transcript of 14 kb. The protein encoded by this transcript is called dystrophin since mutations lead to the Becker and Duchenne muscular dystrophies [1]. DMD is located within a common fragile site (CFS), which is a region of profound genomic instability. CFS regions often comprise genes that are frequently deleted or altered in cancers and act as tumor-suppressor genes [2]. 

Recently, Wang et al. (2014) reported DMD deletions in 25 of 40 myogenic tumors (29 gastrointestinal tumors (GIST), 4 rhabdomyosarcomas, and 7 leiomyosarcomas (LMS)), and observed that forced re-expression of the miniDMD construct (lacking exons 17–48) inhibited cell migration, cell invasion, anchorage independence, and invadopodia formation [3]. The role of DMD as a tumor suppressor in sarcomas is strengthened by the observation that dystrophin-deficient mdx mice, which are murine models for Duchenne muscular dystrophy, spontaneously develop rhabdomyosarcomas in 6–9% of cases [4]. More strikingly, Schmidt et al. (2011) noticed that the mdx mutation led in 39% of cases to the spontaneous formation of skeletal-muscle-derived malignant tumors in mice, presenting as mixed rhabdomyo-, fibro-, and liposarcomas (LPS) [5]. In line with this observation, inactivation of other genes implicated in muscular dystrophy often accompanies age-related soft-tissue sarcomas (STS), thereby suggesting a common mechanism for their oncogenesis (Schmidt et al. 2011). Interestingly, these tumors harbor aneuploidy and genomic instability, which is illustrated by numerous double-strand breaks and the activation of DNA-repair mechanisms, but also recurrent genetic lesions (Trp53, Nf1, Cdkn2a, etc.) that provide links to human mesenchymal cancers and especially to sarcomas with complex genomic profiles [5]. 

However, overexpression of the DMD gene has also been reported in leukemias, renal carcinomas, ependymomas, and astrocytomas [6], which strengthens the argument for its role in oncogenesis. In addition, high DMD expression was associated with poorer overall survival in B-cell chronic lymphocytic leukemia [7]. None of these observations suggest that DMD acts as a tumor suppressor. As a possible way to account for these discrepancies, the DMD locus comprises at least five internal promoters that give rise to shorter dystrophin transcripts, which encode smaller isoforms sharing the same COOH-terminal domains [8]. Each of these promoters initiates the translation of a unique first exon. This leads to the production of different-sized proteins: Dp427 (427 kDa), Dp260 (260 kDa), Dp140 (140 kDa), Dp116 (116 kDa), Dp71 (71 kDa), and Dp45 (45 kDa). Whereas Dp427 is the one that is altered in muscular dystrophies, Dp71 is ubiquitously expressed, except in differentiated muscle cells, and is involved in cognition, cell adhesion, cell cycle, and in nuclear-envelope-related functions [9]. Evidence is accumulating for the versatile role of Dp71 in cancer: on one hand, it seems to act as a tumor-suppressor gene in gastric adenocarcinoma [10], yet on the other, its inhibition decreases the growth and malignancy of the A549 cell line both in vitro and in vivo. Furthermore, Wang et al. (2014) observed that inhibition of Dp71 dramatically decreased the growth of RMS cell lines [3]. They also showed that inhibition of DMD enhanced migration and invasion, whereas its re-expression reduced migration and induced senescence in melanoma cell lines.

To elucidate the oncogenic impact of DMD in sarcoma oncogenesis, we studied the genomic status of DMD in 318 sarcomas, mainly those with complex genomic profiles but alsosynovial sarcomas and GIST. We also investigated isoform expression and performed functional analyses to decipher the role of Dp427 and Dp71, which are the two isoforms expressed in these sarcomas. 

## 2. Results

### 2.1. DMD Deletion Is Associated with Metastatic Progression

In a cohort of 318 STS (103 sarcomas with complex genetic profiles (SCG) comprising 33 leiomyosarcomas (LMS), 23 myxofibrosarcoma, 37 undifferentiated pleomorphic sarcoma (UPS), and 10 others; 88 synovial sarcomas (SS); and 127 GISTs) (Table 1), analyzed by CGH array, *DMD* deletion was detected in 16.5% of all tumors (16.5% of sarcomas with complex genomic profiles, 21.6% of synovial sarcomas, and 14.2% of GIST) (Table 1).

**Figure 1:**
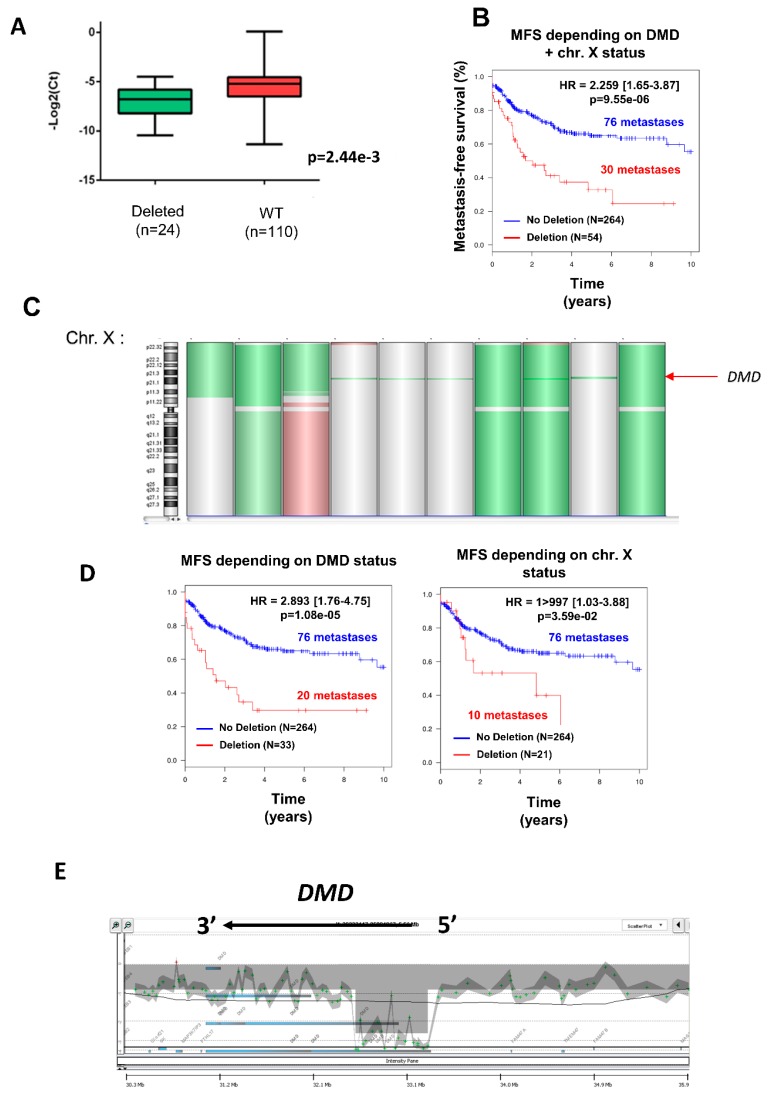
Deletion of DMD gene was significantly associated with metastatic evolution. (**A**) DMD expression determined by TaqMan comparing tumors with (in green) or without (in red) DMD deletion. (**B**) Kaplan–Meier analysis of metastasis-free survival (MFS) of sarcoma patients divided into two groups according to their DMD status (non-deleted in blue, DMD deleted in red). (**C**) Chromosome X (chr. X) analyzed by GCH array: losses and gains are represented in green and red, respectively. Three types of deletion affecting DMD are illustrated: deletion of entire chr. X, of the short arm of chr. X, or targeting only DMD (indicated by a red arrow). (**D**) Kaplan–Meier analysis of metastasis-free survival of sarcoma patients divided into two groups: (in blue) patients displaying no deletion and (in red) patients displaying either targeted DMD deletion (left plot) or deletion of p-arm/entire X chromosome (right plot). (**E**) Zoom on DMD genomic status on a GCH-array profile. Illustration of a typical deletion targeting the DMD gene.

Deletion was significantly associated with a decrease in DMD expression (Figure 1A) (*p* = 0.002), independently of gender (53.7% of men vs. 46.3% of women) (Table 2), but was significantly more frequent in metastatic tumors (Chi^2^ = 2.68 × 10^−4^; Table 2). Indeed, metastasis occurred in 30/54 (55.6%) of deleted tumors against 76/264 (28.8%) in non-deleted tumors. Accordingly, the DMD genomic status split the tumors into two groups with significant distinct prognosis, showing that DMD deletion is associated with a worse prognosis (Figure 1B).

Three different types of deletion could be observed (Figure 1C): the entire X chromosome, the p arm of chromosome X, and within the DMD locus, which was the most frequent (61.1% of cases). To verify that the prognostic value of DMD deletion is not related to large alterations affecting the X chromosome but has a significant relevance by itself, metastasis-free survival (MFS) analysis was compared between non-deleted patients and patients with a targeted deletion of DMD (referred to as “DMD status”) or with a deletion of the p-arm or the entire X chromosome (referred to as “chr. X status”) (Figure 1D). MFS analysis showed that the greatest prognostic value was obtained by the narrowed DMD deletion and not by the loss of the p-arm or the entire X chromosome (Figure 1D), thus confirming that DMD deletion is an independent marker of metastatic evolution.

**Figure 2:**
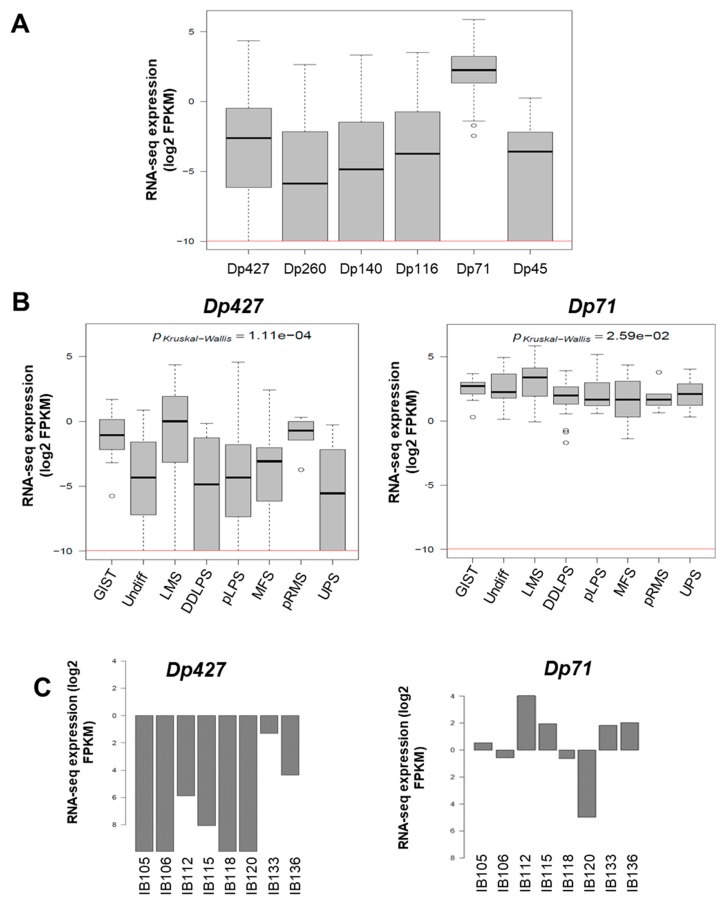

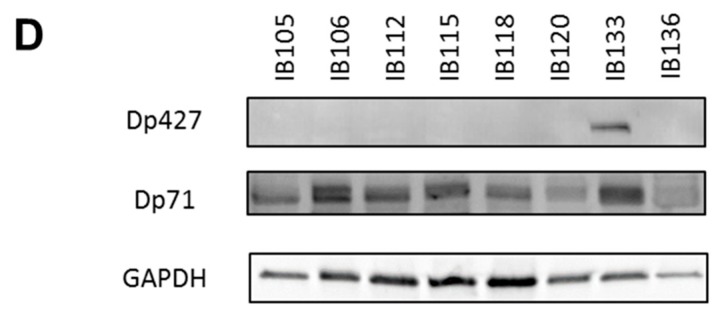
Expression of DMD isoforms in soft-tissue sarcoma (STS) tumors and cell lines. (**A**) Respective expression of DMD isoforms in 145 tumors determined by RNA sequencing. Red horizontal line is minimal expression threshold: 0.001 Fragments Per Kilobase Million (FPKM) ~−9.97 log2 FPKM. (**B**) Expression of Dp427 and Dp71 depending on tumor histotype obtained by RNA sequencing. GIST: gastrointestinal tumor; Undiff: undifferentiated sarcoma; LMS: leiomyosarcoma; DDLPS: dedifferentiated liposarcoma; pLPS: pleomorphic liposarcoma; MFS: myxofibrosarcoma; pRMS: pleomorphic rhabdomyosarcoma; UPS: undifferentiated pleomorphic sarcoma. (**C**) Respective expression of Dp427 and Dp71 in sarcoma cell lines determined by RNA sequencing. (**D**) Respective abundance of Dp427 and Dp71 in sarcoma cell lines assessed by Western blotting.

As illustrated in Figure 1E, DMD deletions were all restricted to the 5’ extremity of the genes, and consequently the 3’ region coding the Dp71 isoform was conserved. To quantify the respective expression of DMD isoforms, we re-analyzed RNA-sequencing data from a cohort of 145 sarcomas previously published by our lab [11]. All DMD isoforms had a low to very low level of expression, except for Dp71, which was significantly the most expressed among tumors (*p* < 0.0001) (Figure 2A). Dp427 expression was restricted to myogenic tumors (GIST, LMS, and pleomorphic RMS), whereas Dp71 was widely expressed across the histotypes (Figure 2B). 

The expression profile was similar in our panel of sarcoma cell lines (which included UPS: IB105 and IB106; LMS: IB112, IB133, IB118, and IB136; and LPS: IB115), with low to very low Dp427 expression and higher Dp71 expression in all cell lines (Figure 2C). This was confirmed by Western blotting, with no Dp427 expression detected, except for the IB133 cell line, but with ubiquitous Dp71 expression in all cell lines (Figure 2D).

### 2.2. Impact of Dp427 and Dp71 on Tumoral Phenotype

**Figure 3:**
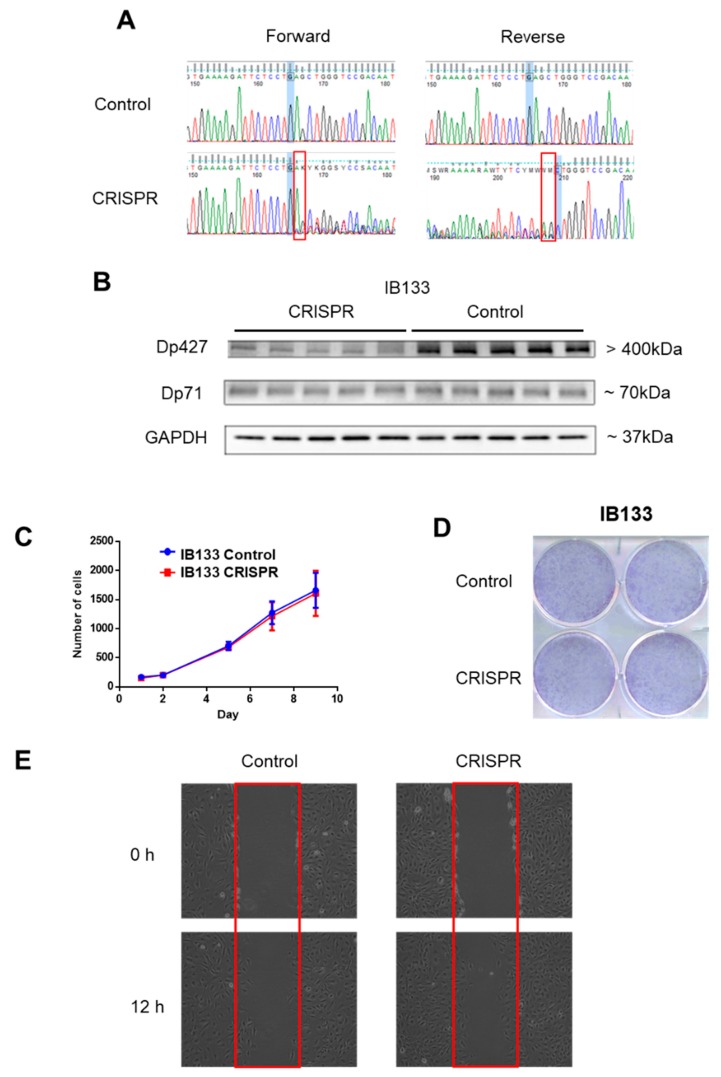
Dp427 downregulation had no impact on cell proliferation or clonogenicity. (**A**) Validation by Sanger sequencing of DMD deletions induced in IB133 cell line after lentiviral transduction of the CRISPR/Cas9 system. (**B**) Validation by Western blot of Dp427 downregulation in cell lines deleted by the CRISPR/Cas9 system. (**C**) Control (in blue) or deleted (at least partially) for Dp427 (in red) cell populations were cultured for 9 days and counted by flow cytometry, with six replicates for each time point. (**D**) Clonogenicity was measured with crystal violet staining and plates were scanned. (**E**) Cells were grown to confluence in six-well plates, and then a “scratch” (wound gap) was created in the cell monolayer using a pipette tip. Images were taken at the moment of the “scratch” (0 h) and 12 h later. Results shown are representative of three independent experiments performed in duplicate.

Since Dp427 was the target of deletions and Dp71 was never altered and was the only one expressed, we studied the impact of each of these isoforms first on metastatic outcome and then on tumor phenotypes. According to RNA sequencing data, metastasis-free survival (MFS) analysis depending on Dp427 or Dp71 expression showed that the isoform expression level was not prognostic of metastatic progression for sarcoma patients (Supplementary Figure S1). This suggests that the deletion of Dp427 is the critical mechanism related to metastatic development, rather than each isoform expression level taken apart. Consequently, Dp427 downregulation was induced by CRISPR/Cas9 editing in the IB133 cell line (derived from leiomyosarcoma), which was selected for its relatively high Dp427 expression at both transcriptomic (Figure 2C) and protein (Figure 2D) levels. Sanger sequencing showed that this CRISPR assay produced the deletion of one or two nucleotides (Figure 3A) associated with Dp427 down-regulation, as evidenced by Western blotting (Figure 3B) in five replicates. Despite effective Dp427 downregulation, we did not observe any significant difference in cell proliferation (Figure 3C), clonogenic properties (Figure 3D), or migration (Figure 3E) between the mutated and control cell lines. 

**Figure 4:**
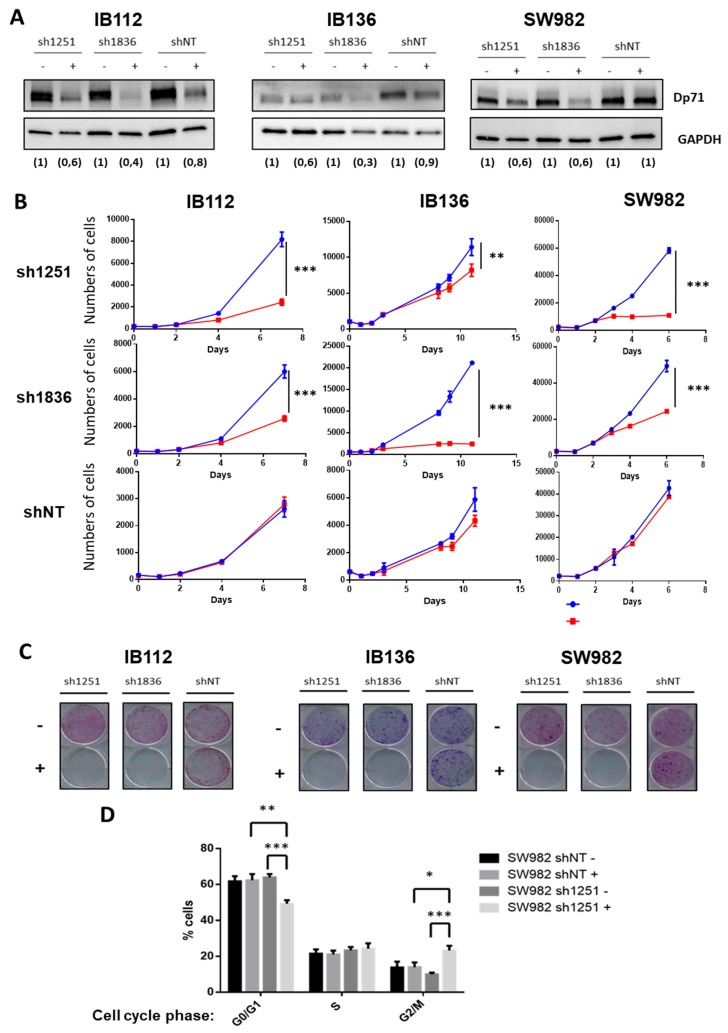
Dp71 inhibition reduced cell proliferation and clonogenicity. (**A**) Effective Dp71 inhibition by sh1251 and sh1836 was verified by Western blotting in IB112, IB136, and SW982 cell lines incubated for 72 h without or with doxycycline. Densitometry quantification is indicated below each lane. GAPDH was used as loading control. (**B**) Cells were counted by flow cytometry with six replicates for each time point. Doxycycline was added (in red) in order to induce Dp71 inhibition, or not (in blue), in culture medium. (**C**) Clonogenicity was measured with crystal violet staining after 12 days of cell culture with (+) or without (−) doxycycline. Then, plates were scanned. (**D**) After 72 h incubation with or without doxycycline, cells were trypsinized, fixed, stained with propidium iodide, and analyzed by flow cytometry. Results shown are representative of three independent experiments performed in duplicate. * *p*-value *p* < 0.05, ** *p* < 0.01, and *** *p* < 0.001.

These observations contrasted with the effect of Dp71 inhibition on one synovial sarcoma (SW982) and two other LMS (IB112 and IB136) cell lines that all expressed Dp71 but not Dp427. Indeed, two shRNA (under the control of the Tet promoter) targeting Dp71 (sh1251 and sh1836) and a control shNT (non-targeting) were transduced. With doxycycline, as validated by Western blotting, effective inhibition of Dp71 was achieved with both shRNA targeting Dp71, as compared to shNT (Figure 4A). Cell count was significantly diminished in all cell lines expressing shRNA 1251 or 1836, but not in those transduced with control shNT (Figure 4B). Of note, Dp71 inhibition even abolished the capacity of cells to grow as isolated clones (Figure 4C). Furthermore, incorporation of propidium iodide (Figure 4D) in SW982-sh1251 vs SW982-shNT, incubated or not during 72 h with doxycycline, showed that Dp71 inhibition reduced the proportion of cells in the G0/G1 phase while increasing the percentage of cells in the G2/M phase, thus suggesting a G2/M-phase arrest. Unfortunately, this result could not be confirmed on the LMS cell lines due to the interference of their intrinsic polyploidy with DNA content analysis. Altogether, these observations demonstrate that Dp71 downregulation dramatically reduces the proliferation of sarcoma cell lines, probably by impairing the progression of cell cycle through G2/M phase as suggested by our observations in SW982 cells.

## 3. Discussion

Pleomorphic sarcomas are characterized more by frequent chromosome losses or gains than by recurrent single nucleotide variations [12]. The deleted genes most frequently reported are p53, RB1 and PTEN, with frequencies ranging from 10% to 50% [12]. Here we report that DMD was deleted in 16.5% of pleomorphic sarcomas with complex genomics (myxofibrosarcoma, UPS, LMS, and pleomorphic liposarcoma (pLPS)), 14.2% of GIST, and 20.7% of synovial sarcomas (SS). These proportions are particularly high in comparison with other studies that report a deletion frequency ranging from 3.4% to 5.5% in melanomas [5] and carcinomas [9]. In contrast, Wang and collaborators observed intragenic deletions in 25 of 40 (63%) high-grade myogenic tumors, which suggests that DMD deletion could be associated with aggressiveness [3]. This is consistent with our data demonstrating that DMD deletions—especially those restricted to this gene—are associated with a poorer prognosis. Some authors have suggested that DMD deletion is restricted to sarcomas with a myogenic differentiation. However, we also noticed such a deletion in myxofibrosarcoma, UPS, pLPS, and SS. This observation may have two major implications. On one hand, these tumors might share a muscular origin, as has been proposed for SS [13]. On the other hand, DMD loss might affect not only skeletal muscle cells but also other mesenchymal tissues. Indeed, DMD-deleted sarcomas could arise from multipotent mesenchymal or muscle-derived stem cells. This hypothesis is strengthened by the study of Schmidt et al. (2011), who reported the spontaneous development of mixed rhabdomyo-, fibro-, and lipo-sarcomas harboring genomic instability in 39% of dystrophic mdx mice (characterized by a DMD mutation) [5]. Whatever the hypothesis, these deletions might arise from anomalies during DMD transcription. Wang et al. also reported that DMD deletions in women affect only the active X chromosome [3] (i.e., the one that is transcribed). 

Indeed, DMD is one of the longest genes (2.2 Mb) in the human genome, and is located within a CFS [14]. CFS are regions of genomic instability that are highly susceptible to homologous recombination in normal cells [15], and have been described as hot spots for chromosomal rearrangements in the initial phase of oncogenesis [16], leading to the amplification of oncogenes and/or inactivation of tumor suppressor genes. This genomic instability can be explained by the collision between transcription and replication complexes of the long genes. Helmrich et al. (2011) noticed that transcription of these long genes starts in the G2/M phase of the cell cycle and extends to the next S phase [17]. This observation has numerous implications: first, the presence of pre-mRNAs on mitotic chromosomes can interfere with proper chromatin condensation and therefore correspond to regions of genomic fragility. Secondly, this means that transcription pausing or stalling of replication forks leads to the creation of stable RNA:DNA hybrids that form R-loops, which are responsible for DNA double-strand breaks and, therefore, for genomic instability [17]. In the same study, they also showed that the frequency of CFS breaks correlates with its transcription level, thereby confirming that DMD deletion is particularly relevant in myogenic sarcomas or more widely in those derived from DMD-expressing cells. Altogether, this could explain why we observed only deletions and no mutation, and especially deletions targeting only the 5’ part of DMD, which accounted for 61% of deleted cases.

In a cohort of 145 sarcomas, Dp427 and Dp71 were the two isoforms that were mainly expressed in tumors, while the DMD-5’ deletion affected only Dp427 but not Dp71. The Dp427 transcript encodes the full-length dystrophin protein, whose altered expression or loss of function is responsible for muscular dystrophies like Duchenne and Becker myopathies [10]. In addition to its implication in muscular dystrophies, evidence is accumulating for a tumor-suppressor role of Dp427 in melanoma, myogenic sarcomas [3,18], and murine models [4]. Regarding Dp71, its involvement in cancer is less clear, with conflicting results depending on the cancer model [10]. In our sarcoma cell line, Dp427 downregulation did not lead to any modification of cell growth or clonogenic capacities. However, this result should be taken cautiously since we did not exactly reproduce the alterations detected in tumors (i.e., large deletions and not deletions of one or two nucleotides). On the other hand, Dp71 inhibition dramatically reduced cell proliferation and clonogenicity in three sarcoma cell lines. This observation is consistent with the study by Villarreal-Silva and collaborators that showed that Dp71 is involved in cell division [19]. They postulated that Dp71 is localized at the mitotic spindle-cleavage furrow and midbody of PC12 cells and binds to lamin B1 and β-dystroglycan, thereby providing proper localization and stability for these cytokinesis multiprotein apparatuses. This supports our observation that Dp71 inhibition seems to induce a cell cycle arrest during the G2/M phase in the SW982 cell line. This is further evidence that Dp71 plays an essential role in cell growth and tumor development in STS. This may explain why it is widely expressed in tumors and why specific DMD deletions systematically preserve the Dp71 reading frame. 

In conclusion, DMD deletion was observed in 15% of this STS cohort and was significantly associated with metastatic evolution. The fact that Dp427 or Dp71 expression level are not prognostic for metastatic progression is not very surprising given that Dp427 and Dp71 are respectively barely and widely expressed among tumors and so, Dp71 or Dp427 expression levels taken apart may not be the determining parameter for metastatic progression. On the other hand, this strengthens the idea that the impact of DMD on metastatic progression is rather determined by the overall expression pattern of the different isoforms—that is to say, Dp71 expression combined with Dp427 deletion—and possibly relies on their protein interactions and/or subcellular localizations. Finally, our work points to Dp71 as a highly interesting therapeutic target, at least in Dp427-deleted sarcoma tumors. This would highlight the point elegantly made by Muller et al. in 2012, when they stated that deletions can expose cancer-specific therapeutic vulnerabilities when the deleted gene is a member of a functionally redundant family of genes carrying out an essential function [20]. 

## 4. Materials and Methods

### 4.1. Ethics Statement

The samples used in this study are part of the Biological Resources Center of Institut Bergonié (CRB-IB). In accordance with the French Public Health Code (articles L. 1243-4 and R. 1243-61), the CRB-IB received agreement from the French authorities to deliver samples for scientific research (number AC-2008-812). Pleomorphic sarcoma, synovial sarcoma, and GIST cohorts have already been reported in previous studies from our team [21,22,23,24]. Every case was histologically reviewed by the pathologist subgroup of the French Sarcoma Group and classified according to the 2013 World Health Organization classification by histology, immunohistochemistry, and molecular genetics and cytogenetics when needed (Cohort description in Table 1).

### 4.2. Cell Lines

Cell lines (IB112 and IB136) were established as previously described [25]. Authentication of cell lines was performed by CGH array and compared with the corresponding original tumor. Sarcoma cell lines were cultured in RPMI 1640 + GlutaMAX I (Life Technologies, Inc., brand of ThermoFisher Scientific, Waltham, MA, USA) supplemented with 10% FCS and 1% penicillin-streptomycin (Life Technologies). The HEK-293T cell line was cultured in DMEM + GlutaMAX I (Life Technologies) supplemented with 10% FCS and 1% penicillin-streptomycin (Life Technologies). Cells were grown at 37 °C in a humidified atmosphere containing 5% CO_2_. 

Sarcoma cell lines IB112, IB136, and SW982 were infected with a lentiviral vector containing the shRNA sequence against DMD under the control of a Tet-on promoter. Control cell lines were established with lentiviral transduction of a vector containing a non-targeting shRNA. VSV-G-pseudotyped lentiviral particles were produced by co-transfection of 293T cells with previous vectors and the compatible packaging plasmids psPAX2 and pVSVg. Cell lines were incubated overnight with lentiviral supernatants and 8 μg/mL polybrene (Sigma H9268). Stably transduced cells were selected with the addition of puromycin (1 µg/mL, Sigma P9620) to culture medium. Downregulation of the protein was verified by Western blotting after induction of shRNA expression by doxycycline (2 µg/mL).

IB133 was infected with a lentiviral vector containing the CRISPR/CAS9 system and the gRNA specifically targeting Dp427 isoforms (Hs0000003709, Sigma Aldrich, St. Quentin Fallavier, France). A control cell line was established with lentiviral transduction of a vector containing a gRNA CNeg targeting no sequence of the human genome (CRISPR/CAS gRNA-NEGATIVE CONTROL 1, CRISPR06, Sigma Aldrich). Cell infection was done as previously. Lentiviral transduction of the CRISPR/Cas9 system was performed in five replicates that consequently produced five IB133 cell lines potentially carrying DMD deletion and five control cell lines (Cneg). Deletion and inhibition of Dp427 were verified by Sanger sequencing and Western blot, respectively. 

### 4.3. Bioinformatics Analysis Pipeline for Genomic and Transcriptomic Data

Genomic DNA was extracted using the standard phenol-chloroform extraction protocol [24]. The Affymetrix SNP array 6.0 (Affymetrix, Santa Clara, CA, USA) was used according to the manufacturer’s instructions. One hundred and six samples were normalized with the Genotyping console 2.0 software (Affymetrix). Total RNAs were extracted as previously described [21]. RNA quality was checked on an Agilent 2100 Bioanalyzer (Agilent Technologies, Santa Clara, CA, USA). Samples were then analyzed on Human Genome U133 Plus 2.0 array (Affymetrix), according to the manufacturer’s procedures. All expression data obtained are publicly available on https://www.ncbi.nlm.nih.gov/geo/query/acc.cgi?acc=GSE71118.

### 4.4. RNA Sequencing

The process from RNA extraction to final BAM files was previously described [11]. We used SAMtools and BCFtools (v0.1.19) [26,27] with custom depths (at least two alternate bases and five total bases) for reporting a candidate variant. These variants were then annotated by ANNOVAR (October 2013) [28] with the hg19 genome version, transcriptome annotations (November 2013), and using the databases of the observed variants: dbSNP (v138) [29], 1000G project (April 2012) [30], ClinVar (September 2014) [31], and COSMIC (v70) [32].

### 4.5. Sanger Sequencing

To observe the effect of CRISPR/Cas9 on gDNA, the primers used were: CATTGAAAGCTAGAAGGTGAG (Forward) and GTTCATTCCAATGGAACGTTAG (Reverse). The Touchdown 60 °C program was used (TD 60 °C; two cycles at 60 °C, followed by two cycles at 59 °C, two cycles at 58 °C, three cycles at 57 °C, three cycles at 56 °C, four cycles at 55 °C, four cycles at 54 °C, five cycles at 53 °C, and finally 10 cycles at 52 °C). PCR was performed on 25 ng of DNA using AmpliTaqGold DNA polymerase (Applied Biosystems, brand of ThermoFisher Scientific, Waltham, MA, USA). PCR products were then purified using the ExoSAP-IT PCR purification kit (GE Healthcare), and sequencing reactions were performed with the Big Dye Terminator V1.1 kit (Applied Biosystems) according to the manufacturer’s recommendations. Samples were then purified using the Big Dye XTerminator purification kit (Applied Biosystems) according to the manufacturer’s instructions, and sequencing was performed on a 3130xl Genetic Analyzer (Applied Biosystems). Sequence analysis was performed with SeqScape software v2.5 (Applied Biosystems).

### 4.6. Western Blot

Cells were rinsed with ice-cold PBS and lysed for 20 min at 4 °C in RIPA lysis and extraction buffer (R0278, Sigma) supplemented with a protease/phosphatase inhibitor cocktail (11697498001, Roche, Basel, Switzerland). Lysates were pelleted for 10 min at 15,000× *g* at 4 °C and supernatants were collected for protein quantitation (DC protein assay kit, Bio-Rad, Hercules, CA, USA). The total proteins of each sample (40 µg) were loaded on gels and separated by SDS-PAGE. Following transfer onto a PVDF membrane using a dry transfer system, membranes were blocked in non-fat dry milk in PBS-Tween 0.1% and then incubated with the primary antibody: Mouse anti-Dp427 (NCL-DYS1, Leica Biosystem, Nussloch, Germany) or Rabbit anti-Dp71 (Ab15277, Abcam) at 4 °C overnight. After washing, blots were incubated for 1 h with a horseradish-peroxidase-linked anti-rabbit antibody (Amersham, brand of GE Healthcare Europe GmbH, Velizy-Villacoublay, France) and processed for chemiluminescent substrate (Amersham ECL Select detection reagent kit, Sigma) according to the manufacturer’s instructions. Signal was detected using the Fusion Fx7 (Thermo Fisher Scientific, Waltham, MA, USA) imaging system. GAPDH (Santa Cruz Biotechnology, sc-166574, Dallas, TX, USA) was used as a loading and the quantification of protein abundance was performed by blot densitometry using the ImageJ 1.48v software (Rasband, W.S., ImageJ, U. S. National Institutes of Health, Bethesda, MD, USA).

### 4.7. TaqMan Expression

cDNAs were synthesized from 1 μg of RNA using the GeneAmp RNA PCR core kit (Applied Biosystem, Courtaboeuf, France). Quantitative PCR analyses were performed using TaqMan Assays-on-demand Gene expression reagents (Applied Biosystem) with qPCR Mastermix Plus without UNG (Eurogentec, Belgium). We used the TaqMan Gene Expression assays provided by Applied Biosystems. The assay IDs were as follows: Hs00758098_m1 for DMD, and Hs99999902_m1 for RPLP0. To normalize the results, we used the RPLP0 gene as a reference gene.

### 4.8. Proliferation Assay

For this, 5000 cells were seeded in five replicates in 96-well plates. Culture medium was changed every 2 days with or without doxycycline. Every 2 to 3 days, cells were washed, trypsinized, and harvested in a final volume of 200 µL of PBS1X plus 10% of FBS. The number of viable cells was evaluated by flow cytometry (FACS Calibur, BD Biosciences, San Jose, CA, USA) based on their morphological features. Data were acquired using CellQuestPro software (BD FACS Systems, Sunnyvale, CA, USA) and analyzed using FlowJo (Tree Star, Celeza GmbH, Ashland, OR, USA) and GraphPad (La Jolla, CA, USA) software.

### 4.9. Clonogenic Assay

To assess cell clonogenic activity, 1000 cells were plated on 6-well plates in standard culture medium. Cells were incubated for 10 days at 37 °C and 5% of CO2. Medium was changed every 2 days with or without doxycycline. Then, cells were washed with PBS 1X, fixed for 5 min with 70% ethanol and then stained with crystal violet and scanned.

### 4.10. Migration Assay

For the wound healing assay, 4.105 cells were plated on a 6-well plate. Twenty-four hours later, a strip of cells was removed from the monolayer of cells using a pipette tip. Phase contrast images were acquired with a 10× objective at the time of the scratch and 12 hours later using a Nikon Eclipse TS100 microscope.

### 4.11. Cell Cycle

Cells were fixed after 48 h incubation with or without doxycycline. Then, cells were stained with propidium iodide with the FxCycle™ PI/RNase Staining Solution (F10797, Life Technologies) according to the manufacturer’s instructions. Data were acquired using CellQuestPro software (BD FACS Systems, Sunnyvale, CA, USA) and at least 10,000 cells per sample were analyzed using FlowJo (Tree Star, Celeza GmbH).

### 4.12. Statistical Analysis

Metastasis-free survival (MFS) was defined as the interval between diagnosis and the time of distant recurrence or the last follow-up. Survival rates were estimated using the Kaplan–Meier method and compared using the log-rank test and hazard ratios (HRs). 

Each experiment was repeated at least three times. To examine the statistical significance of the results, analyses were performed with Prism6 v6.01 (GraphPad software Inc., La Jolla, CA, USA) software. Normal distribution of data sets was examined with a Shapiro–Wilk normality test. If data passed the test, the statistical significance between two conditions was assessed with an unpaired *t*-test, and results were represented as mean ± standard deviation (SD). Otherwise, a Mann–Whitney test was used. Significant differences are represented as * *p*-value *p* < 0.05, ** *p* < 0.01, and *** *p* < 0.001.

## 5. Conclusions

In conclusion, we observed that DMD was frequently deleted in soft tissue sarcomas and that this deletion was significantly associated with metastatic progression. Although Dp427 is the main target of these deletions, our in vitro data suggest that Dp71 is essential for tumor maintenance, at least by promoting cell proliferation. This also raises Dp71 as a novel potential therapeutic target for sarcoma care.

## Figures and Tables

**Table 1:** Cohort description. SCG: sarcomas with complex genetic profiles GIST: gastrointestinal tumor; SS: synovial sarcoma.

Cohort	SCG (n = 103)	GIST (n = 127)	SS (n = 88)
Median follow-up (years)	2.05 (1.44–2.93)	3.60 (3.01–4.18)	2.61 (1.80–3.12)
Median age at diagnosis (years)	63 (59–66)	64 (59–68)	28 (22–36)
Gender			
Male	53 (51%)	64 (50%)	48 (55%)
Female	50 (49%)	63 (50%)	40 (45%)
Metastasis	35 (34%)	38 (30%)	33 (38%)
Relapse	26 (25%)	10 (8%)	16 (18%)
Grading system	FNCLCC:	AFIP:	FNCLCC:
	Grade 1: 8 (8%)	Very low: 14 (11%)	Grade 1: 1 (1%)
	Grade 2: 25 (24%)	Low: 15 (12%)	Grade 2: 23 (26%)
	Grade 3: 68 (66%)	Intermediate: 79 (62%)	Grade 3: 61 (69%)
	Unknown: 2 (2%)	High: 18 (14%)	Unknown: 3 (3%)
		Unknown: 1 (1%)	
DMD Loss	17 (16.5%)	18 (14.2%)	19 (21.6%)

**Table 2:** Distribution of DMD deletion depending on gender or metastasis.

	DMD Loss	Normal DMD	Chi^2^ *p*-Value
Gender			0.881
Male (n = 165)	29	136	
Female (n = 153)	25	128	
Metastasis			2.68 × 10^−4^
Yes (n = 106)	30	76	
No (n = 212)	24	188	
Total	54	264	

## Supplementary Materials

The following are available online at https://www.mdpi.com/2072-6694/11/7/922/s1, Figure S1: Kaplan Meier analysis of metastasis-free survival of 149 sarcoma patients, Figure S2: Signal detection on the whole membrane shown on Figure 2D, Figure S3: Signal detection on the whole membrane shown on Figure 3D, Figure S4: Signal detection on the whole membranes shown on Figure 4A.
