# Supplementary material for: Recurrent DMD Deletions Highlight Specific Role of Dp71 Isoform in Soft-Tissue Sarcomas
Source: Cancers (Basel). 2019 Jul 1;11(7):922. doi: 10.3390/cancers11070922 (PMC6678178; doi:10.3390/cancers11070922)
Supplement: Supplementary file 1 [file cancers-11-00922-s001.pdf]

Supplementary Material

Recurrent DMD Deletions Highlight Specific Role of Dp71 Isoform in Soft-Tissue Sarcomas

Olivier Mauduit, Vanessa Delcroix, Tom Lesluyes, Gaëlle Pérot, Pauline Lagarde, Lydia Lartigue, Jean-Yves Blay and Frédéric Chibon

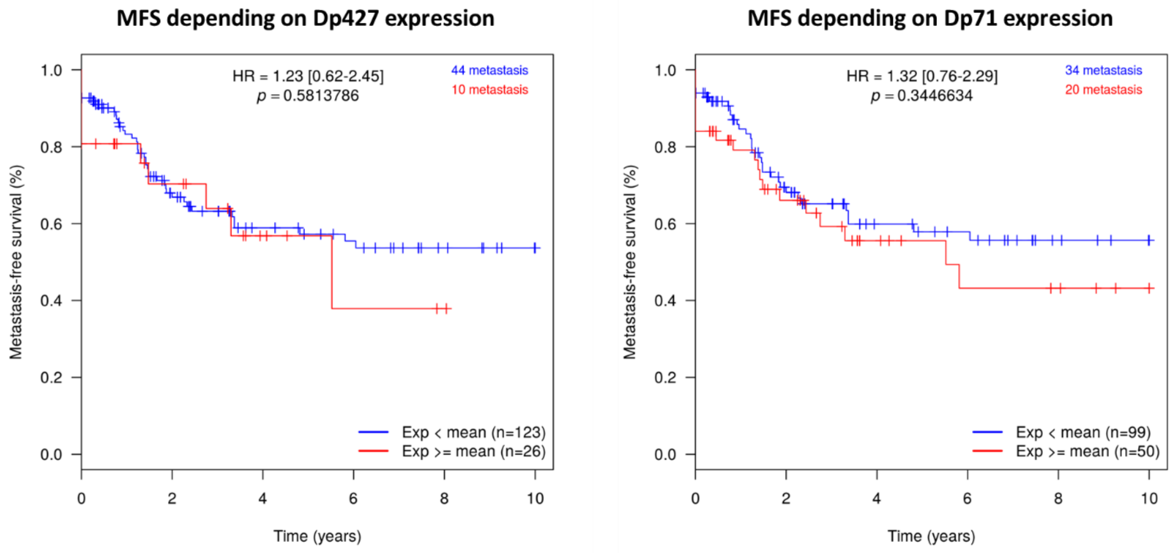

Supplementary Figure S1. Kaplan Meier analysis of metastasis-free survival of 149 sarcoma patients divided into two groups according to Dp427 (left panel) or Dp71 (right panel) expression level (low and high in blue and red, respectively) determined by RNA sequencing.

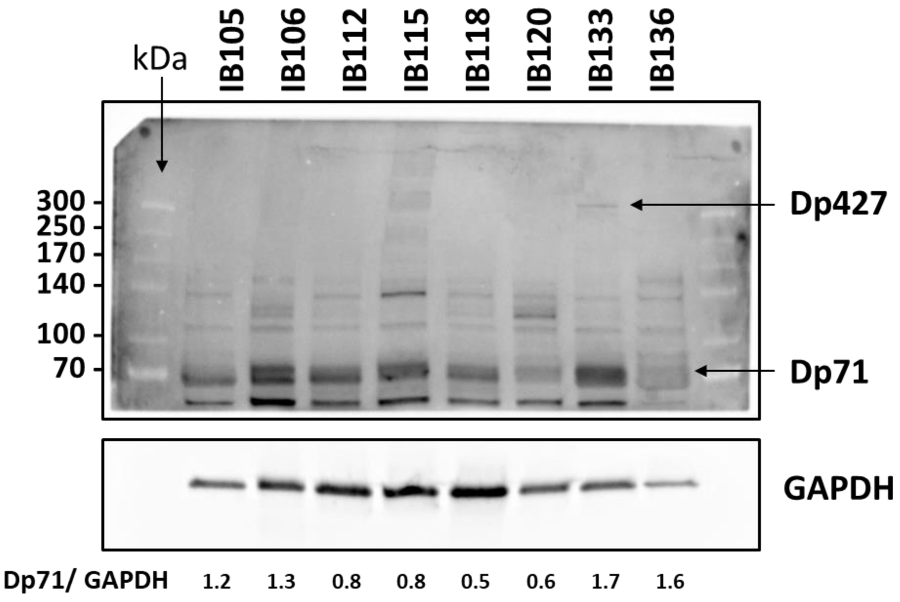

Supplementary Figure S2. Signal detection on the whole membrane shown on Figure 2D. Dp427 and Dp71 expression in sarcoma cell lines assessed by western blotting. GAPDH was used as loading control.

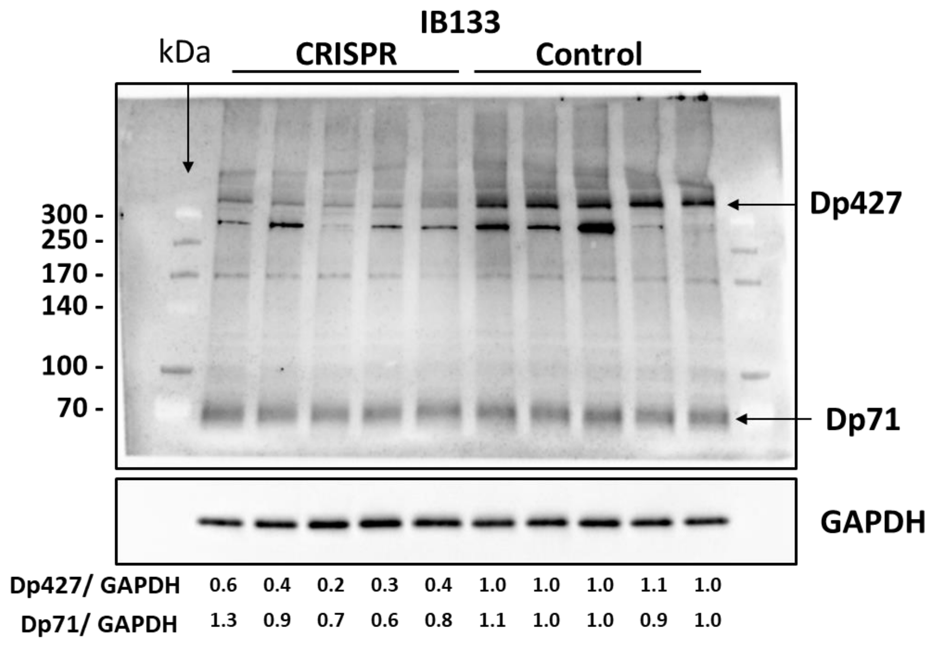

Supplementary Figure S3. Signal detection on the whole membrane shown on Figure 3D. Dp427 and Dp71 expression in sarcoma cell lines assessed by western blotting. GAPDH was used as loading control. To compare isoforms expression between cell lines, results were normalized to the mean of the control group set to 1.

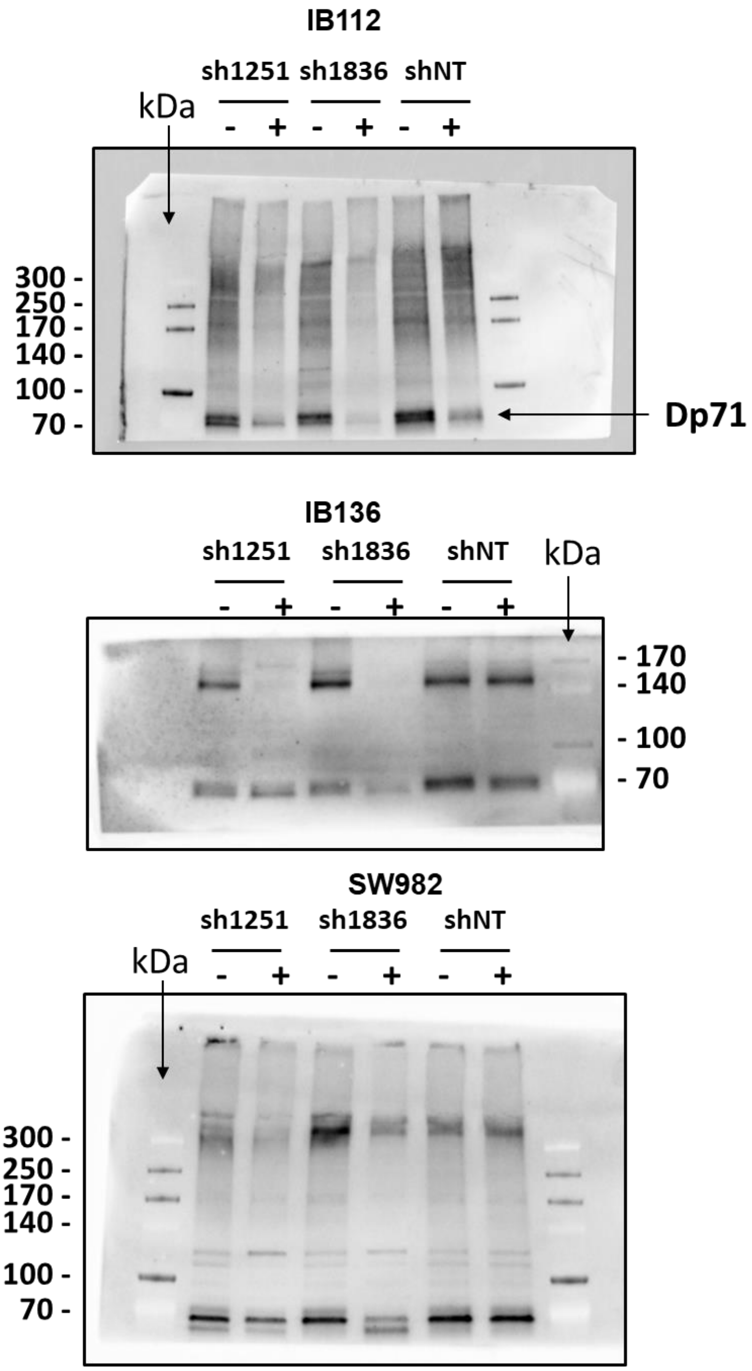

Supplementary Figure S4. Signal detection on the whole membranes shown on Figure 4A. Dp71 expression in sarcoma cell lines assessed by western blotting. Quantification of Dp71 expression is displayed on Figure 4A, below panels.
